# Comparison of outcomes for HLA-matched sibling and haplo-identical donors in Myelodysplastic syndromes: report from the chronic malignancies working party of EBMT

**DOI:** 10.1038/s41408-022-00729-y

**Published:** 2022-09-28

**Authors:** Kavita Raj, Dirk-Jan Eikema, Vipul Sheth, Linda Koster, Liesbeth C. de Wreede, Didier Blaise, Carmela Di Grazia, Yener Koc, Victoria Potter, Patrice Chevallier, Lucia Lopez- Corral, Depei Wu, Stephan Mielke, Johan Maertens, Ellen Meijer, Anne Huynh, Jakob Passweg, Thomas Luft, Jose Antonio Pérez-Simón, Fabio Ciceri, Agnieszka Piekarska, G. Hayri Ozsan, Nicolaus Kröger, Marie Robin, Ibrahim Yakoub-Agha

**Affiliations:** 1 University College London NHS Foundation Trust, London, UK; 2 EBMT statistical Unit, Leiden, The Netherlands; 3 Fred Hutchinson Cancer Research Centre Seattle, Seattle, WA USA; 4 EBMT Data Office Leiden, Leiden, The Netherlands; 5 Biomedical Data Sciences, LUMC, Leiden, The Netherlands; 6 Programme de Transplantation &Therapie Cellulaire, Marseille, France; 7 Ematologia e terapie cellulari. IRCCS Ospedale Policlinico San Martino, Genova, Italy; 8 Medicana International Hospital, Istanbul, Turkey; 9 Kings College Hospital, London, UK; 10 CHU Nantes, Nantes, France; 11 Hospital Clínico, Salamanca, Spain; 12 First Affiliated Hospital of Soochow University, Suzhou, China; 13 Karolinska University Hospital, Stockholm, Sweden; 14 University Hospital Gasthuisberg, Leuven, Belgium; 15 VU University Medical Center, Amsterdam, The Netherlands; 16 CHU - Institut Universitaire du Cancer Toulouse, Toulouse, France; 17 University Hospital, Basel, Switzerland; 18 University of Heidelberg, Heidelberg, Germany; 19 Hospital Universitario Virgen del Rocío, Instituto de Biomedicina de Sevilla (IBIS / CISC), Universidad de Sevilla, Sevilla, Spain; 20 Ospedale San Raffaele s.r.l., Milano, Italy; 21 Medical University of Gdansk, Gdańsk, Poland; 22 Dokuz Eylul University, Izmir, Turkey; 23 University Hospital Eppendorf, Hamburg, Germany; 24 Hopital St. Louis, Paris, France; 25 CHU de Lille, Univ Lille, INSERM U1286, Infinite, 59000 Lille, France

**Keywords:** Stem-cell research, Myelodysplastic syndrome

## Abstract

Myelodysplastic syndromes (MDS) are the second common indication for an Allo-HCT. We compared the outcomes of 1414 matched sibling (MSD) with 415 haplo-identical donors (HD) transplanted with post-transplant cyclophosphamide (PTCy) as GVHD prophylaxis between 2014 and 2017. The median age at transplant with MSD was 58 and 61 years for HD. The median time to neutrophil engraftment was longer for HD being 20 vs 16 days for MSD (*p* < 0.001). Two-year overall survival (OS) and PFS (progression free survival) with MSD were significantly better at 58% compared with 50%, *p* ≤ 0.001, and 51% vs 47%, *p* = 0.029, with a HD. Relapse at 2 years was lower with a HD 23% than with MSD 29% (*p* = 0.016). Non relapse mortality (NRM) was higher with HD in the first 6 months post-transplant [HR 2.59 (1.5–4.48) *p* < 0.001] and was also higher at 2 years being 30% for HD and 20% for MSD, *p* ≤ 0.001. The incidence of acute GVHD grade II-IV and III–IV at 100 days was comparable for MSD and HD, however, chronic GVHD at 2 years was significantly higher with MSD being 44% vs 32% for HD (*p* < 0.001). After multivariable analysis, OS and primary graft failure were significantly worse for HD particularly before 6 months [HR 1.93(1.24–3.0)], and HR [3.5(1.5–8.1)]. The median age of HD 37 (IQR 30–47) years was significantly lower than sibling donors 56 (IQR 49–62 years) *p* < 0.001. However, there was no effect on NRM, relapse or PFS. This data set suggests that a MSD donor remains the preferred choice in MDS over a haplo donor. Transplants with haploidentical donors result in satisfactory long-term outcome, justifying it’s use when no better donor is available.

## Introduction

The myelodysplastic syndromes are a heterogenous cluster of clonal stem cell disorders that occur in the older adult manifesting as either bone marrow failure and or a progression towards acute leukemia. Allogeneic hematopoietic cell transplantation (allo-HCT) is the only option that offers the potential for long-term disease-free survival in 30–50% of recipients [1–4]. Both conditioning intensity and donor type affect outcomes. A prospective French study showed similar outcomes in patients who received allo-HCT from an HLA-matched sibling donor (MSD) with those from an HLA-matched unrelated donor (10/10) [5]. A CIBMTR analyses confirmed these results when compared matched 8/8 unrelated donors to those from MSD albeit with higher non relapse mortality [RR 1.44 (95% CI 1.06–1.95)] [5, 6]. More recently the donor pool has been extended to the use of haploidentical donors (HD) particularly utilizing T-replete stem cells with post-transplant cyclophosphamide (PTCy) GVHD (graft versus host disease) prophylaxis [7]. This trend was confirmed in an analysis of HD for MDS in Europe wherein outcomes were improved with reduced intensity conditioning and the use of PTCy as GVHD prophylaxis [8]. A further comparison of HD with PTCy and mismatched unrelated/cord blood (MMUD/CB) donors showed lower non-relapse mortality, acute GVHD, and better overall survival for HD when compared to both MMUD and CBD [9, 10]. In older recipients with MDS/AML who were transplanted with MUD or Haplo donor, overall survival was similar with lower GVHD among Haploidentical recipients [11]. Recipient age, however, remains the most important prognostic factor predicting outcomes in HD transplant [12]. Recently, a comparison of Haplo stem cell transplants in patients with acute leukemia from sibling or offspring donors showed that in patients younger than 55, the outcomes from MSD were similar to the Haplo recipients conditioned with PTCy [13]. Conversely older recipients who predominantly had offspring donors had higher graft failure, NRM and overall mortality [14, 15] compared to MSD [13]. Given that the median age of presentation of MDS is in the 6th–7th decade and haplo-HCT across the EBMT registry has been increasing [16], we sought to compare the outcomes of MSD and an alternative albeit family HD, and also address the debatable issue as to whether readily available younger family mismatched donor should be a preferred choice over older MSD in older patients receiving allo-HCT [17, 18].

## Methods

All patients provided informed consent for data registration, according to the Declaration of Helsinki. This study was approved by the Chronic Malignancies Working Party of EBMT. Data were retrieved from the EBMT registry for sibling and mismatched family donor transplants performed between 2014 and 2017. Within the mismatched family donor transplants all patients received post-transplant cyclophosphamide as GVHD prophylaxis.

Patients were included if the family donor was ≥2 Ag mismatch and at least Haplotype (3/6) matched. Neutrophil engraftment was defined as the time at which the absolute neutrophil count was >0.5 × 10^9^/L for three consecutive days and platelet engraftment as a platelet count >20 × 10^9^/L for seven consecutive days without transfusion support. Primary graft failure (PGF) was defined as failing to reach neutrophil >0.5 × 10^9^/L in the first 28 days post-transplant or documentation of autologous reconstitution by chimerism analysis in the absence of relapse [19]. Secondary graft failure was defined by the treating physician: standard criteria across Europe would be loss of a functioning graft demonstrated by cytopenia in at least two lineages and loss of donor chimerism. Complete remission (CR) was defined if all the following were achieved: Hb > 11 g/dl, Platelet > 100 × 10^9^/L and Neutrophils >1.5 × 10^9^/L with <5% blast in the bone marrow. Relapse was defined as loss of CR. For this study CR and relapse were designated by the treating physician. Conditioning regimes were defined as myeloablative conditioning (MAC) if they contained either total body irradiation (TBI) with a dose of >6 Gy, oral Busulfan dosage >8 mg/kg or a dose of intravenous Busulfan >6.4 mg/kg. Additional variables in the analyses included remission status, stem cell source, donor gender, donor-recipient gender match and recipient age. Pre-transplant patient characteristics were expressed as the median and interquartile range (IQR) for continuous variables and frequencies and proportions for categorical variables. Primary endpoints were overall survival (OS), progression free survival (PFS), cumulative incidence of relapse/progression and non-relapse mortality (NRM), evaluated until 24 months after transplant. Median follow-up was determined using the reverse Kaplan–Meier method. The cumulative incidences of grade II–IV and III–IV acute GvHD (aGvHD) and limited/extensive chronic GvHD (cGvHD) were also estimated at 100 days and 24 months after the date of engraftment of evaluable patients respectively. GvHD and relapse free survival (GRFS) was estimated at 24 months and was defined as time from engraftment until the first event among grade III–IV aGvHD, extensive cGvHD, relapse and death [20]. The cumulative incidences of neutrophil and platelet engraftment were estimated at 28 days and 100 days, respectively. OS and PFS were estimated using the Kaplan–Meier product limit estimation method, and differences in subgroups until 24 months were assessed by the Log-Rank test. Cumulative incidences of relapse and NRM were analyzed together in a competing risks framework. Competing risks analyses were also separately applied to estimate aGvHD and graft loss, each with competing event death, and cGvHD in which limited cGvHD, extensive cGvHD and death were considered competing events. For neutrophil engraftment and platelet engraftment, the competing events were graft loss, relapse and death before any of these events. Subgroup differences were assessed using Gray’s test. All estimates were reported with 95% confidence intervals. All *p*-values were two-sided and *p* < 0.05 was considered significant. Statistical analyses were performed using SPSS version 22 (SPSS Inc., Chicago, IL) and R version 3.0.3 (R core team, Vienna, Austria) using packages ‘prodlim’, ‘survival’ and ‘cmprsk’.

## Results

### Patient characteristics

Patients and transplant characteristics are summarized in Table 1. 1829 patients identified with MDS from 270 transplant centers who underwent either a MSD or HD transplant, (MSD *n* = 1414 (77.3%) and HD, *n* = 415 (22.7%)) were analyzed for outcomes. The median follow-up was 28 (26–30) months. HD recipients were older (HD- 61 years vs MSD 58 years, *p* = 0.001), had WHO higher risk disease (HD—79% vs MSD—70%, *p* < 0.001), higher number of patients not in remission-(40% MSD and 53% HD *p* < 0.001), longer median interval from diagnosis to transplant 12 months vs 8 months for MSD (*p* = 0.002), had poorer Karnofsky performance status (KPS) with 65% having a KPS of 90–100% compared to 71% for MSD *p* = 0.033, had more patients having received male to female mismatched grafts (*p* = 0.001), and having CMV seropositivity (*p* = 0.01, 77% vs 70%). HD donors were significantly younger (37 years vs 56 years, *p* = <0.001). The data to complete IPSS score including cytogenetics was largely unavailable (Table 1). Donor characteristics are described in detail in supplementary text.

**Table 1:** Demographic profile of population.

		Donor				
	Group	MSD		HD		*p*
		Missing	*N* (%)	Missing	*N* (%)	
Total			1414 (77.3%)		415 (22.7%)	
Donor age (years)	Median (IQR)	369 (26.1%)	55.6 (49–62.2)	57 (13.7%)	36.7 (29.4–46.8)	<0.001
Patient age (years)	Median (IQR)		57.9 (49.9–63.6)		60.9 (52.2–66.2)	0.001
Patient sex	Male		869 (61.5%)		269 (64.8%)	0.2
	Female		545 (38.5%)		146 (35.2%)	
Sex match	Female to male	12 (0.8%)	410 (29.2%)		85 (20.5%)	0.001
	Other combinations		992 (70.8%)		330 (79.5%)	
WHO classification	RA/RARS/del5q	62 (4.4%)	74 (5.5%)	26 (6.3%)	13 (3.3%)	<0.001
	RCMD-(RS)		210 (15.5%)		45 (11.6%)	
	RAEB-1		260 (19.2%)		53 (13.6%)	
	RAEB-2		446 (33%)		154 (39.6%)	
	Transformed to AML		244 (18%)		96 (24.7%)	
	MDS Unclassifiable		118 (8.7%)		28 (7.2%)	
Interval diagnosis-HCT	Median (IQR)		7.8 (4.6–16.7)		11.6 (6.1–24.8)	0.002
Disease status at HCT	CR	68 (4.8%)	446 (33.1%)	16 (3.9%)	109 (27.3%)	<0.001
	no CR		532 (39.5%)		213 (53.4%)	
	Untreated		368 (27.3%)		77 (19.3%)	
Stem cell source	BM		127 (9%)		160 (38.6%)	<0.001
	PB		1272 (90%)		254 (61.2%)	
	BM + PB		15 (1.1%)		1 (0.2%)	
Infused CD34 (10^6^/Kg)	Median (IQR)	935 (66.1%)	5 (3.6–6.7)	261 (62.9%)	5 (3.4-6.7)	0.3
CMV serostatus patient	Negative	27 (1.9%)	422 (30.4%)	6 (1.4%)	97 (23.7%)	0.01
	Positive		965 (69.6%)		312 (76.3%)	
Conditioning intensity	Standard	17 (1.2%)	582 (41.7%)	2 (0.5%)	199 (48.2%)	0.022
	Reduced		815 (58.3%)		214 (51.8%)	
TBI	No	16 (1.1%)	1250 (89.4%)	2 (0.5%)	319 (77.2%)	<0.001
	Yes		148 (10.6%)		94 (22.8%)	
In-vivo T cell depletion*	No	2 (0.1%)	768 (54.4%)		378 (91.1%)	<0.001
	Yes		644 (45.6%)		37 (8.9%)	
ATG	No	2 (0.1%)	877 (62.1%)		378 (91.1%)	<0.001
	Yes		535 (37.9%)		37 (8.9%)	
Alemtuzumab	No	2 (0.1%)	1303 (92.3%)		415 (100%)	<0.001
	Yes		109 (7.7%)			
PTCy	No	33 (2.3%)	1324 (95.9%)			<0.001
	Yes		57 (4.1%)		415 (100%)	
Karnofsky score	<90	89 (6.3%)	383 (28.9%)	20 (4.8%)	137 (34.7%)	0.033
	90–100		942 (71.1%)		258 (65.3%)	

*MSD* matched sibling donor, *CR* complete remission, *haplo* haploidentical donors, *TBI* total body irradiation, *PTCY* post-transplant cyclophosphamide, *ATG* anti-thymocyte globulin, *IQR* interquartile range, *HSCT* Haematopoeitic stem cell transplant, *CMV* cytomegalovirus, *BM* bone marrow, *PB* peripheral blood, *PB* peripheral blood *In vivo T cell depletion excluding PTCY.

### Engraftment

Neutrophil engraftment at 28 days was higher and faster for MSD transplants (*p* < 0.001) being 95% (95% CI 94–96%), median time 16 (95% CI 16–17) days vs 80% (95% CI 76–84%), median time of 20 (95% CI 19–20) days with HD (see Table 2). Neutrophil engraftment by D28 was positively related with use of PB graft (93% (92–94%) versus 82% (78–87%), *p* < 0.001), RIC (92% (90–94%) versus 91% (87–92%), *p* = 0.011), no PTCy (95% (94–97%) versus 81% (77–85%), *p* < 0.001) and being in CR at transplant (94% (92–96%) versus 88% (86–90%), *p* < 0.001). Similarly, the incidence of platelet engraftment at 100 days was higher with MSD recipients 94% (95% CI 93–96%), median time of 14 (95% CI 14–15) days vs 75% (95% CI 71–80%) median time of 28 (27–31 days) in HD recipients (*p* < 0.001). Platelet engraftment was positively related with PB (91% (89–92%) versus 86% (81–90%), *p* < 0.001) graft but inversely with PTCy (77% (73–81%) versus 94% (93–96%), *p* < 0.001). The delay in neutrophil and platelet engraftment in HD compared to MSD seems primarily due to different donor matching—MSD vs HD rather than the stem cell source being PB or BM as there was no difference in engraftment in HD recipients based on the graft source (Supplemental Table 1).

**Table 2:** Outcomes as per donor source in univariate analysis.

	MSD	HD	*p*
**ANC engraftment (day 28)**	95% (94–96%)	80% (76–84%)	*p* < 0.001
**Median (95% CI)**	16 (16–17)	20 (19–21)	
**Platelet engraftment (day 100)**	94% (93–96%)	75% (71–80%)	*p* < 0.001
**Median (95% CI)**	14 (14–15)	28 (27–31)	
**Primary graft failure (2** **yr)**	2% (1–2%)	10% (7–12%)	p < 0.001
**Secondary graft failure (2** **yr)**	4% (3–5%)	3% (2–5%)	0.8
**aGvHD II-IV (day 100)**	21% (19–24%)	24% (20–28%)	0.3
**aGvHD III–IV (day 100)**	10% (8–11%)	10% (7–13%)	0.7
**cGvHD (2** **yr)**	44% (41–47%)	32% (28–37%)	*p* < 0.001
**Limited cGvHD (2** **yr)**	23% (20–25%)	14% (10–18%)	*p* < 0.001
**Extensive cGvHD (2** **yr)**	18% (15–20%)	17% (14–21%)	0.9
**OS (2** **yr)**	58% (55–61%)	50% (45–55%)	*p* < 0.001
**PFS (2** **yr)**	51% (48–54%)	47% (42–53%)	0.029
**Relapse (2** **yr)**	29% (26–32%)	23% (18–27%)	0.016
**NRM (2** **yr)**	20% (17–22%)	30% (25–35%)	*p* < 0.001
**GRFS (2** **yr)**	26% (23–29%)	31% (25–37%)	0.9

*MSD* matched sibling donor, *haplo* haploidentical donor, *ANC* absolute neutrophil counts, *HCT* Haematopoeitic cell transplant, *GVHD* graft vs host disease, *OS* overall survival, *GRFS* GVHD free relapse free survival, *NRM* non-relapse mortality, *yr* year, *PFS* progression free survival.

Primary graft failure was more frequent in HD recipients 10% (95% CI 7–12%) vs 2% (95% CI 1–2%) of MSD recipients (*p* < 0.001). Secondary graft failure was similar for both MSD 4% (95% CI 3–5%) and HD 3% (2–5%) *p* = 0.8.

### Survival and relapse/GVHD

**Fig. 1:**
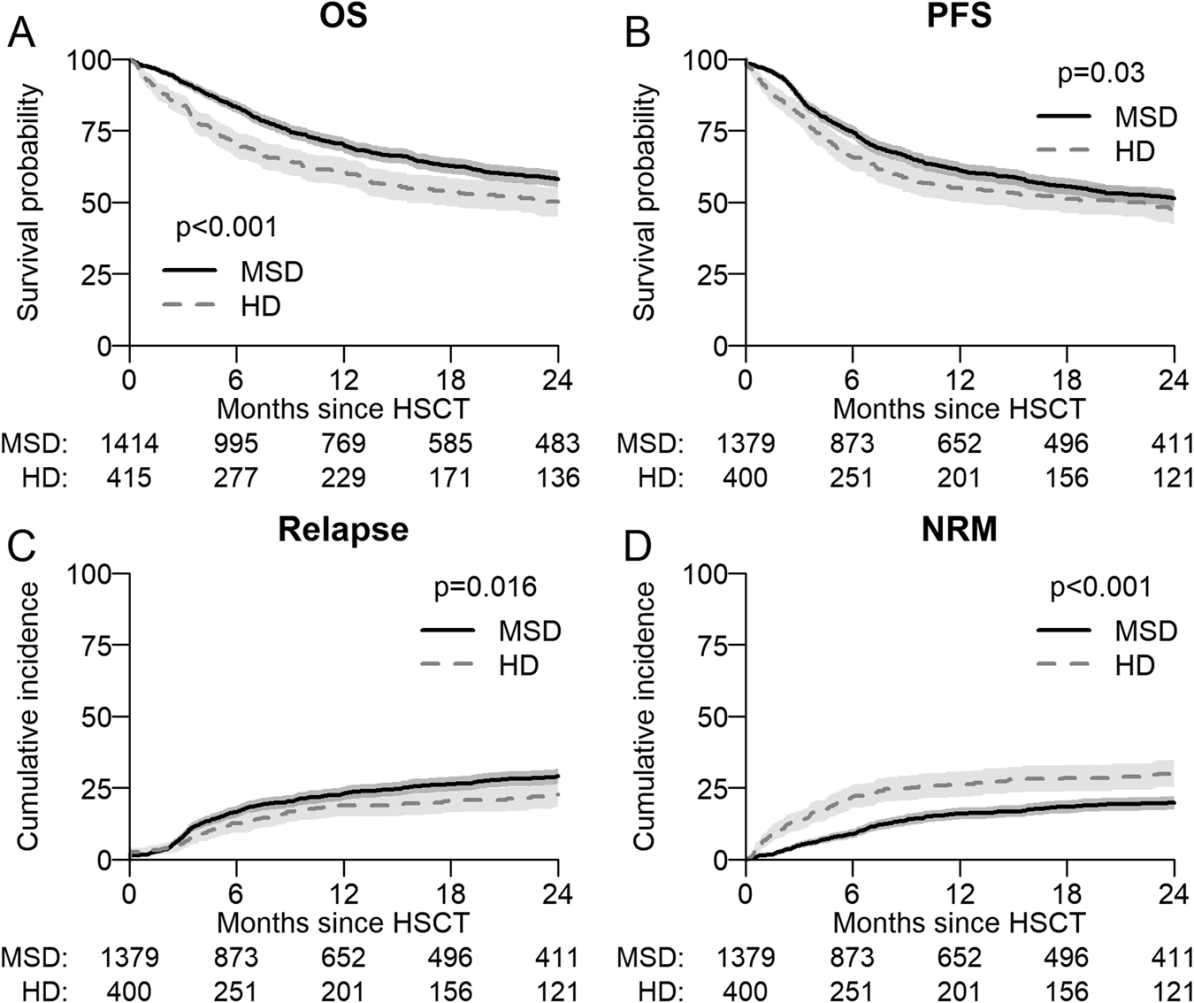
Survival probability and cumulative incidence of relapse. **A** Overall Survival (OS) and **B** Progression Free Survival (PFS) post-transplant of entire cohort as estimated by the method of Kaplan–Meier. **C** Cumulative Incidences of relapse (CIR) and **D** Non-Relapse Mortality (NRM) for entire cohort.

**Fig. 2:**
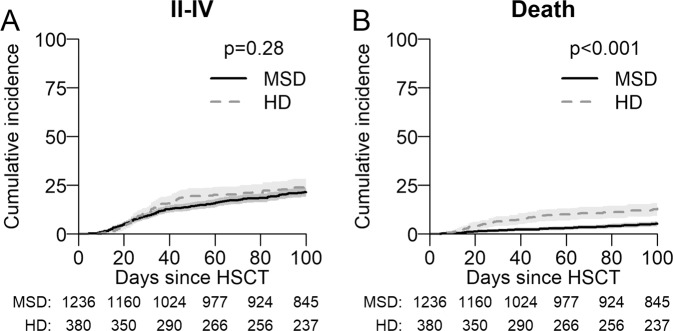
Cumulative Incidence of aGVHD. **A** Cumulative incidence of acute Graft Versus Host Disease (aGvHD) grade II–IV. **B** Cumulative incidence of death due to aGVHD.

**Fig. 3:**
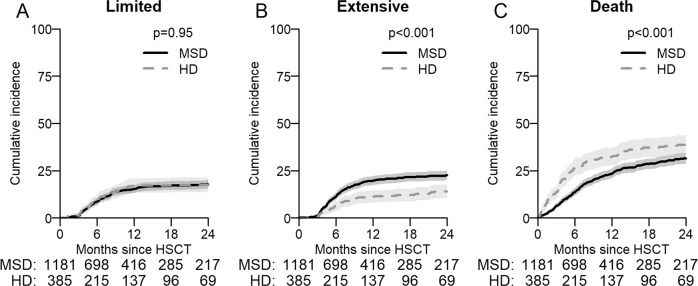
Cumulative incidence of cGHVD. **A** Cumulative incidence of limited chronic GvHD until 2 years after transplant. **B** Cumulative incidence of extensive chronic GvHD until 2 years after transplant. **D** Cumulative incidence of death without cGvHD, **C** cumulative incidence of death due to cGVHD.

The median follow-up was 28 months (95% CI 26–30) and all patients included had sufficient data to estimate relapse incidence. While OS, PFS at 24 months was significantly better in recipients of a MSD compared to HD (Table 2, Fig. 1A–D) the incidence of relapse at 2 years was lower in patients with a HD at 23% (95% CI 18–27%) vs 29% (CI-26–32%) with MSD *p* = 0.016. Relapse/disease progression as a cause of mortality occurred more frequently with MSD (137 patient: 29% vs 35 18% for HD). GVHD free relapse free survival (GFRS) at 24 months was similar between sibling 26% (95% CI 23–29%) and HD 31% (95% CI 25–37%) *p* = 0.9 (supplementary Fig. 1). There was no difference in the incidence of aGVHD (100 days) however cGVHD at 2 years, largely limited cGHVD, occurred more frequently with MSD 44% (95% CI 41–47%) than with HD 32% (28–37%) *p* < 0.001 (Table 2, Figs. 2, 3). In this study HD tend to fail from GRFS from early events (mostly death), whereas sibling donors tend to fail from later (non-terminal) events such as extensive cGvHD and relapse. Unfortunately, GRFS does not differentiate between these event types.

### Non-relapse mortality

NRM was higher with HD being 30% (95% CI 25–35%) vs 20% (95% CI 17–22%) at 2 years with MSD (*p* < 0.001) (Table 2). The most frequent cause of NRM was infection in HD 74 (39%) versus 119 (25%) with MSD, whereas death from GHVD was similar (111 patients, 23% of patients with MSD and 38 patients (20%) with Haplo donor). The adverse effect of a HD occurred particularly in the first 6 months post-transplant with 112 deaths. Infection caused 58/112 deaths (48% of all deaths in HD within 6 months, majority 25% being bacterial followed by 10% fungal). Between six and 24 months there were no differences in the incidences of causes of death between MSD and HD.

### Multivariable analysis

The results of multivariable analysis are summarized in Table 3. Primary graft failure was significantly higher for haplo as compared to MSD [HR = 3.56 (95%CI-1.56–8.17; *p* = 0.003)]. The use of HD resulted in poorer OS [HR = 1.93 (95% CI 1.24–3, *p* = 0.004)] particularly in the first 6 month due to a higher NRM [HR = 2.59, (95% CI-1.5-4.5; *p* < 0.001)] than MSD whereas this was similar after the first 6 months HR 0.93 (95% CI 0.42–2.07, *p* = 0.9). PFS was similar [HR = 1.16 (95%CI 0.77–1.76; *p* = 0.5)], as was relapse [HR = 0.56 (95% CI 0.29–1.1; *p* = 0.09)]. Disease status, utilizing the WHO classification, with either excess blasts or AML at transplant did not influence OS, NRM, PFS or relapse. For recipients treated prior to a transplant, the absence of CR was associated adversely with OS [HR = 1.31 (95% CI 1.07–1.6; *p* = 0.009)], but not PFS [HR = 1.16 (95% CI 0.95–1.4; *p* = 0.14)] or relapse [HR = 1.04 (95% CI 0.81–1.34; *p* = 0.73)]. Both untreated and patients not in CR experienced increased NRM HR 1.45 (95% CI 0.99–2.11) *p* = 0.056 and 1.41 (95% CI 1.03–1.93) *p* = 0.0315 respectively. Surprisingly, untreated patients had a lower risk of relapse [HR = 0.52 (95% CI 0.36–0.74; *p* < 0.001)] when compared to those who received treatment (whether in CR or not in CR). PB as a stem cell source was not associated with higher NRM [HR = 1.25 (95% CI 0.87–1.8; *p* = 0.2)]. The use of donor other than female to male was associated with lower NRM [HR 0.7 (95%CI 0.54–0.91; *p* = 0.009)], and higher relapse [HR = 1.37 (1.06–1.78; *p* = 0.016)] (Table 3). On multivariate analysis HD was the significant factor for death from infection (*p* < 0.001). Causes of death overall, within six and between 6 and 24 months and multivariate analysis are summarized in supplementary table 1A–D, and Supplementary Fig. 2.

**Table 3:** Multivariate analysis of factors affecting Outcomes. A Multivariate analysis for factors affecting OS. B Multivariate analysis of factors affecting NRM. C Multivariate analysis of factors affecting PFS. D Multivariate analysis of factors affecting relapse. E Multivariate analysis of factors affecting graft failure.

A	Group	HR (95% CI)	*p*
Donor (HD vs MSD)	<6 m	1.93 (1.24–3)	0.004
	>6 m	0.74 (0.44–1.26)	0.3
Donor age (dec)	<6 m	1 (0.84–1.19)	0.96
	>6 m	1.19 (1.02–1.4)	0.026
Donor x donor age	<6 m	0.99 (0.78–1.25)	0.9
	>6 m	0.76 (0.59–0.98)	0.034
Patient age (dec)		1.04 (0.94–1.16)	0.5
WHO classification	RA/RARS/del5q/RCMD-(RS)		
	RAEB-1/2	0.97 (0.75–1.25)	0.8
	Transformed to AML	1.28 (0.95–1.73)	0.11
	Missing	0.86 (0.6–1.24)	0.4
Disease status at HSCT	CR		
	No CR	1.31 (1.07–1.6)	0.009
	Untreated	0.85 (0.65–1.12)	0.3
Stem cell source	BM		
	PB	1.25 (0.97–1.6)	0.088
CMV serostatus patient	Negative		
	Positive	1.25 (1.03–1.52)	0.026
TBI	No		
	Yes	1.03 (0.8–1.33)	0.8
Sex match	Female to male		
	Other combinations	0.88 (0.73-1.07)	0.2
Conditioning intensity	Standard		
	Reduced	1.03 (0.85–1.26)	0.7

B	Group	HR (95% CI)	*p*
Donor (HD vs MSD)	<6 m	2.59 (1.5–4.48)	<0.001
	>6 m	0.93 (0.42–2.07)	0.9
Donor age (dec)	<6 m	0.99 (0.76–1.29)	0.9
	>6 m	0.91 (0.63–1.33)	0.6
Donor x Donor age	<6 m	0.88 (0.64–1.2)	0.4
	>6 m	0.8 (0.53–1.19)	0.3
Patient age (dec)		1.08 (0.93–1.25)	0.3
WHO classification	RA/RARS/del5q/RCMD-(RS)		
	RAEB-1/2	1 (0.7–1.42)	>0.99
	Transformed to AML	1.1 (0.71–1.71)	0.7
	Missing	0.71 (0.41–1.21)	0.2
Disease status at HCT	CR		
	No CR	1.41 (1.03–1.93)	0.031
	Untreated	1.45 (0.99–2.11)	0.056
Stem cell source	BM		
	PB	1.25 (0.87–1.8)	0.2
CMV serostatus patient	Negative		
	Positive	1.4 (1.04–1.88)	0.026
TBI	No		
	Yes	0.83 (0.56–1.21)	0.3
Sex match	Female to male		
	Other combinations	0.7 (0.54–0.91)	0.009
Conditioning intensity	Standard		
	Reduced	0.97 (0.73–1.28)	0.8

C	Group	HR (95% CI)	*p*
Donor (HD vs MSD)	<6 m	1.16 (0.77–1.76)	0.5
	>6 m	0.71 (0.4–1.25)	0.2
Donor age (dec)	<6 m	1.05 (0.91–1.22)	0.5
	>6 m	1.19 (1.01–1.4)	0.037
Donor x Donor age	<6 m	0.88 (0.71–1.09)	0.2
	>6 m	0.83 (0.63–1.09)	0.17
Patient age (dec)		1.04 (0.94–1.16)	0.5
WHO classification	RA/RARS/del5q/RCMD-(RS)		
	RAEB-1/2	0.96 (0.75–1.22)	0.7
	Transformed to AML	1.27 (0.95–1.7)	0.11
	Missing	0.89 (0.63–1.25)	0.5
Disease status at HCT	CR		
	No CR	1.16 (0.95–1.4)	0.14
	Untreated	0.82 (0.64–1.06)	0.13
Stem cell source	BM		
	PB	1.13 (0.89–1.44)	0.3
CMV serostatus patient	Negative		
	Positive	1.26 (1.05–1.52)	0.015
TBI	No		
	Yes	1.01 (0.8–1.29)	0.9
Sex match	Female to male		
	Other combinations	1.01 (0.84–1.22)	0.9
Conditioning intensity	Standard		
	Reduced	1.07 (0.89–1.28)	0.5

D	Group	HR (95% CI)	*p*
Donor (HD vs MSD)	<6 m	0.56 (0.29–1.1)	0.09
	>6 m	0.56 (0.25–1.27)	0.17
Donor age (dec)	<6 m	1.03 (0.85–1.25)	0.7
	>6 m	1.12 (0.9–1.39)	0.3
Donor x Donor age	<6 m	0.87 (0.64–1.19)	0.4
	>6 m	0.86 (0.58–1.26)	0.4
Patient age (dec)		1.02 (0.88–1.18)	0.8
WHO classification	RA/RARS/del5q/RCMD-(RS)		
	RAEB-1/2	0.92 (0.66–1.29)	0.6
	Transformed to AML	1.39 (0.94–2.05)	0.097
	Missing	1.02 (0.65–1.6)	0.9
Disease status at HCT	CR		
	No CR	1.04 (0.81–1.34)	0.7
	Untreated	0.52 (0.36–0.74)	<0.001
Stem cell source	BM		
	PB	1.02 (0.74–1.42)	0.9
CMV serostatus patient	Negative		
	Positive	1.17 (0.92–1.5)	0.2
TBI	No		
	Yes	1.19 (0.87–1.62)	0.3
Sex match	Female to male		
	Other combinations	1.37 (1.06–1.78)	0.016
Conditioning intensity	Standard		
	Reduced	1.17 (0.91–1.5)	0.2

E			Primary graft failure		Secondary graft failure	
		*N*	HR (95% CI)	*p*	HR (95% CI)	*p*
Donor	MSD	988				
	HD	334	3.56 (1.56–8.17)	0.003	1.15 (0.55–2.4)	0.7
Conditioning intensity	Standard	518				
	Reduced	804	0.9 (0.48–1.69)	0.7	0.87 (0.49–1.56)	0.6
Stem cell source	BM	205				
	PB	1117	0.71 (0.36–1.41)	0.3	0.61 (0.31–1.21)	0.16
Patient age (decades)		1322	1.08 (0.81–1.44)	0.6	0.9 (0.69–1.18)	0.5
Donor age (decades)		1322	0.81 (0.61–1.06)	0.13	1.21 (0.93–1.58)	0.15

*TBI* total body irradiation, *CMV* cytomegalovirus, *OS* overall survival, *PFS* progression free survival, *NRM* non-relapse mortality, *BM* bone marrow, *PB* peripheral blood.

### Outcomes based on donor and recipient age

**Fig. 4:**
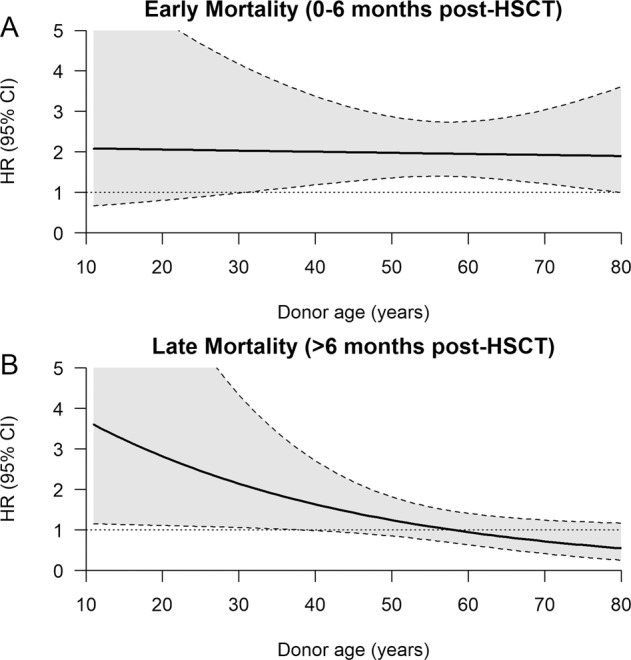
Hazard ratio of mortality when comparing HD vs MSD donors of the same age. **A** HR for OS according to donor age comparison of haploidentical with sibling donor within 6 months (**B**) after 6 months.

Increasing donor age (sibling or haplo) by decade in general appeared not to influence overall survival in the first 6 months but increasing donor age had a detrimental effect on OS after 6 months [HR = 1.19 (95%CI (1.02–1.4); *p* = 0.026)] Table 3a. The effect of donor age from a HD compared to a MSD shows (Fig. 4A) that there is no effect of donor age in the first 6 months in either MSD or HD group as the dominant effect on survival was primarily related to receiving a HD transplant. However, Fig. 4B shows that after 6 months younger HD when compared to younger MSD have a higher mortality (HR > 1) whereas older HD appear to have a similar effect on mortality as MSD of the same age. There was no effect of HD age on PFS, NRM or relapse (Fig. 4A, B). Recipient age did not impact on OS [HR = 1.04 (95% CI .94–1.16; *p* = 0.5)], PFS [HR = 1.04 (95% CI 0.94–1.16; *p* = 0.5)] or NRM [HR = 1.08 (95% CI 0.93–1.25; *p* = 0.3)] in either HD or MSD groups (Table 3). We further examined a subgroup of recipient ≥40 years, and we found no difference in survival and overall outcomes for this group by donor type (Supplementary Table 3).

## Discussion

With the limited availability of MSD and increased feasibility of performing HD transplants [16] comparison of outcomes using haplo donors with MSD are needed to guide donor selection [12, 17, 18]. Furthermore, for the patients with MDS it is unclear whether we can extrapolate from reports focused on AML, regarding selection between an older MSD or a younger HD (easily available for all patients) transplanted with PTCy [13, 18, 21, 22]. Our data from a recent cohort of patients with MDS who underwent Haploidentical transplants with post-transplant cyclophosphamide show that results for HD are inferior to MSD transplants due to the increased NRM, especially in the first 6 months.

In comparison with MSD, as expected, the relapse rate with HD is relatively lower. This may be due to a higher graft versus leukemia effect attributed to HD, as has also recently been shown by Robin et al- relapses being comparable between mismatched MUD, HD and cord blood transplantation [9]. In our study, The WHO disease category RAEB1/RAEB2/AML did not have any effects on relapse/PFS. However, disease status (response to chemotherapy- absence of CR) was independently associated with worse PFS and relapse rates. This is in keeping with previous studies showing that disease related risk factors mainly affect outcomes [14]. The IPSS-R, EBMT transplant specific risk score for MDS, GITMO and CIBMTR scores correlate well with the risk of relapse [23–25], as also does disease risk index (DRI) rather than the type of donor [14, 23, 24, 26]. Of note, we witnessed significantly lower relapse rates and NRM for transplant from female to male donor which probably might be related to enhanced graft vs leukemia effects without GVHD (non-tolerized donor T cells) against SMCY, which might have translated into survival/PFS benefit [27, 28]. We found lower relapse rates among untreated patients as compared to patients who received treatment (irrespective of response and disease status-blast count), and this might be due to inability of treatment such as azacytidine to eliminate founder precursor clones, and emergence/selection of resistant clones while on treatment [29–32].

In the current data set, the NRM was significantly higher with HD versus MSD, although there has been a substantial fall in rates of NRM (30%) as compared to previous CMWP EBMT- reports (36–40%). Within the HD cohort graft rejection rates were high at 10% which may significantly contribute to the increased early NRM due to infection, in the first 6 months. NRM was independently predicted by refractory disease, CMV seropositivity of patient and donor recipient sex mismatch (male to female) but did not depend on recipient age. This is in discordance with previous reports [33]- where HCT-CI (co-morbidity index) and recipient age were independently shown to affect NRM [23]. Refractory disease status, on the other hand, has been shown to be detrimental in most of the studies [34]. Given the enhanced NRM with HD, this probably might be a significant variable to be considered while planning conditioning intensity and in vivo depletion strategies for allo-HCT. The implementation of Letermovir prophylaxis could probably help in reducing the incidence of CMV disease and infection rates especially in HD settings [35–38].

The cohort described here were older with a median age of 58 for sibling and 60 for Haplo recipients which is expected for MDS. Within this spectrum, recipient age was not predictive of overall outcomes in both HD and MSD cohorts, unlike previous studies [12, 18]. The effect of increasing donor age by decade was detrimental to overall survival for both HD and MSD. Early mortality however was higher in the HD group irrespective of donor age. For recipient surviving beyond 6 months from transplant, younger HD when compared to a similar age-MSD demonstrate an adverse effect on OS whereas older HD donors had similar outcomes to age matched MSD (Fig. 4B). As the median age of HD 37 (30–47) years was significantly lower than MSD 56 (49–62 years) *p* < 0.001, given the older median age of recipients, the younger HD are likely to be offspring rather than siblings. We hypothesis that the lower survival with younger HD after 6 months may be due their higher graft failure. Robinson et al, similarly described in acute leukemia that the older recipient (>55 years) with a younger offspring donor combination experienced higher graft failure, NRM and lower overall survival compared to the older recipient with a matched MSD donor [13]. Other studies also report that recipients >40 years, especially above 55 years having a younger mismatched offspring donor tend to have a higher rate of NRM and graft rejection and lower overall survival [14, 15] Our data seems to concur, with older recipients experiencing better outcomes with a MSD followed by a mismatched older (likely sibling) donor but poorer outcomes with a mismatched younger donor (likely offspring donor). Unfortunately, we do not have the exact donor relationships and status regarding donor specific antibodies. In addition, younger donors for MSD will always have comparatively younger recipients, but for HD group younger donors could potentially have older or younger recipients (more likely older) and recipient age per se could also have a bearing on overall outcomes.

The existing literature is inconsistent, as another study showed that in recipients >55 years with older MSD, tend to have an increased NRM, and lower PFS and overall survival thus having overall outcomes comparable to Haplo transplants from a younger mismatched family donor [18]. This study, quite contrary to our findings, suggested that given a situation where a choice between a young HD and an older matched donor is to be made, there is a survival benefit of selecting young, HD donor. Lower and delayed neutrophil and platelet engraftment in HD was apparent with primary non engraftment being 10% and significantly higher as compared to (2%) MSD. Although more HD recipient received a BM graft compared to matched MSD donors, the data show that there was no difference in primary neutrophil or platelet engraftment times with either BM or PB in the HD PTCy setting. In patients with acute leukemia there was a suggestion of higher graft failure with BM [39], whereas comparisons in patients having mixed bag of diseases, there was no difference in engraftment between the two types of graft [34, 40].

The study suffers from various drawbacks in that there was inadequate data on cytogenetic risk to inform more precisely the outcomes with IPSS or R-IPSS scores. The incidence of donor directed antibodies also would have helped interpret the non-engraftment data. Importantly valuable information on the kinship of the donors is missing. Additionally, the platforms for transplanting MSD were different to that of the uniform PTCy platform for HD and may influence outcomes. We also do not have data on immune- reconstitution and CMV reactivation, particularly for HD transplants. It could be hypothesized that the HD transplants were at higher risk of NRM in first 6 months probably due to a patient selection bias (higher risk disease) and delay in time to transplantation. Maybe as physicians now are likely to go for earlier HD transplants this may need to be re-evaluated in the future.

These data suggest that matched siblings are the optimal donor in MDS, however in their absence, despite the higher early NRM and primary graft failure, HD transplantation is a reasonable option. Studies in MDS, that include donor kinship to confirm whether a mismatched sibling donor may be preferable to a younger mismatched offspring donor are needed.

## Supplementary information

Supplementary Table 1: Comparison of engraftment by BM or PB for Haplo donor

Supplementary table 2- causes of death

Supplementary table 3- Multivariate analysis for various factors affecting a- OS, b- NRM for patients > or =40 years of age

Supplementary figure 1

Supplementary figure 2

## Data Availability

Database of patient data is available with the corresponding author on request.
