# Supplementary material for: Comparison of outcomes for HLA-matched sibling and haplo-identical donors in Myelodysplastic syndromes: report from the chronic malignancies working party of EBMT
Source: Blood Cancer J. 2022 Sep 28;12(9):140. doi: 10.1038/s41408-022-00729-y (PMC9515068; doi:10.1038/s41408-022-00729-y)
Supplement: Supplementary file 1 — Supplementary Table 1: Comparison of engraftment by BM or PB for Haplo donor [file 41408_2022_729_MOESM1_ESM.docx]

|  | BM | PB | p |
| --- | --- | --- | --- |
|  | HD-BM | HD PB | p |
| ANC engraftment (day 28) | 77% (71-84%) | 82% (77-87%) | 0.16 |
| Median (95% CI) | 20 (20 - 21) | 20 (19 - 21) |  |
| Platelet engraftment (day 100) | 77% (69-85%) | 75% (69-81%) | 0.7 |
| Median (95% CI) | 31 (27 - 34) | 28 (26 - 30) |  |
| Primary graft failure (2yr) | 10% (5-15%) | 9% (6-13%) | 0.8 |
| Secondary graft failure (2yr) | 4% (1-8%) | 3% (1-5%) | 0.4 |
| aGvHD II-IV (day 100) | 13% (8-19%) | 30% (25-36%) | <0.001 |
| aGvHD III-IV (day 100) | 5% (1-9%) | 14% (9-18%) | 0.01 |
| cGvHD (2yr) | 29% (21-36%) | 35% (29-41%) | 0.15 |
| Limited cGvHD (2yr) | 12% (6-18%) | 15% (10-20%) | 0.3 |
| Extensive cGvHD (2yr) | 17% (11-23%) | 18% (13-23%) | 0.7 |
| OS (2yr) | 52% (44-60%) | 49% (43-56%) | 0.4 |
| PFS (2yr) | 45% (37-53%) | 49% (43-56%) | 0.8 |
| Relapse (2yr) | 29% (22-37%) | 18% (13-23%) | 0.03 |
| NRM (2yr) | 26% (19-33%) | 32% (26-38%) | 0.14 |
| GRFS (2yr) | 37% (28-46%) | 28% (21-35%) | 0.01 |

Supplementary Table 1: Comparison of engraftment by BM or PB for Haplo donor
