# Supplementary material for: Comparison of outcomes for HLA-matched sibling and haplo-identical donors in Myelodysplastic syndromes: report from the chronic malignancies working party of EBMT
Source: Blood Cancer J. 2022 Sep 28;12(9):140. doi: 10.1038/s41408-022-00729-y (PMC9515068; doi:10.1038/s41408-022-00729-y)
Supplement: Supplementary file 2 — Supplementary table 2- causes of death [file 41408_2022_729_MOESM2_ESM.docx]

|  |  | donor |  |  |  |  |
| --- | --- | --- | --- | --- | --- | --- |
|  | Group | MSD |  | HD |  | p |
|  |  | Missing | N (%) | Missing | N (%) |  |
| Death | Relapse/progression | 19 (3.9 %) | 137 (29.1 %) | 4 (2.1 %) | 35 (18.4 %) | 0.012 |
|  | Secondary malignancy/PTLD |  | 8 (1.7 %) |  | 2 (1.1 %) |  |
|  | GVHD |  | 111 (23.6 %) |  | 38 (20 %) |  |
|  | Infection |  | 119 (25.3 %) |  | 74 (38.9 %) |  |
|  | Organ damage/failure |  | 33 (7 %) |  | 19 (10 %) |  |
|  | Toxicity |  | 9 (1.9 %) |  | 4 (2.1 %) |  |
|  | Transplant-related death |  | 17 (3.6 %) |  | 9 (4.7 %) |  |
|  | Cell therapy-related |  | 1 (0.2 %) |  |  |  |
|  | Other |  | 35 (7.4 %) |  | 9 (4.7 %) |  |

**Overall causes of death within 2 years- supplementary table 1a**

|  |  | donor |  |  |  |  |
| --- | --- | --- | --- | --- | --- | --- |
|  | Group | MSD |  | HD |  | p |
|  |  | Missing | N (%) | Missing | N (%) |  |
| Death | Relapse/progression | 2 (0.9 %) | 48 (22.1 %) | 1 (0.8 %) | 11 (9 %) | 0.003 |
|  | Secondary malignancy/PTLD |  | 5 (2.3 %) |  |  |  |
|  | GVHD |  | 55 (25.3 %) |  | 23 (18.9 %) |  |
|  | Infection |  | 63 (29 %) |  | 58 (47.5 %) |  |
|  | Organ damage/failure |  | 19 (8.8 %) |  | 16 (13.1 %) |  |
|  | Toxicity |  | 5 (2.3 %) |  | 3 (2.5 %) |  |
|  | Transplant-related death |  | 10 (4.6 %) |  | 6 (4.9 %) |  |
|  | Other |  | 12 (5.5 %) |  | 5 (4.1 %) |  |

**Causes of death within 6 months- Supplementary table 1b**

|  |  | donor |  |  |  |  |
| --- | --- | --- | --- | --- | --- | --- |
|  | Group | MSD |  | HD |  | p |
|  |  | Missing | N (%) | Missing | N (%) |  |
| Death | Relapse/progression | 17 (6.3 %) | 89 (35.2 %) | 3 (4.2 %) | 24 (35.3 %) | 0.95 |
|  | Secondary malignancy/PTLD |  | 3 (1.2 %) |  | 2 (2.9 %) |  |
|  | GVHD |  | 56 (22.1 %) |  | 15 (22.1 %) |  |
|  | Infection |  | 56 (22.1 %) |  | 16 (23.5 %) |  |
|  | Organ damage/failure |  | 14 (5.5 %) |  | 3 (4.4 %) |  |
|  | Toxicity |  | 4 (1.6 %) |  | 1 (1.5 %) |  |
|  | transplant-related death |  | 7 (2.8 %) |  | 3 (4.4 %) |  |
|  | Cell therapy-related |  | 1 (0.4 %) |  |  |  |
|  | Other |  | 23 (9.1 %) |  | 4 (5.9 %) |  |

**Supplementary table 1c-** **Causes of death 6-24 months**

|  |  | n | d | HR (95% CI) | p |
| --- | --- | --- | --- | --- | --- |
| In vivo t cell depletion | no | 792 | 98 |  |  |
|  | yes | 509 | 48 | 0.91 (0.62-1.36) | 0.7 |
| Donor age |  | 1301 | 146 | 0.93 (0.79-1.09) | 0.4 |
| Donor type | MSD | 966 | 86 |  |  |
|  | HD | 335 | 60 | 2.08 (1.32-3.28) | 0.002 |
| Stage of disease | CR | 416 | 36 |  |  |
|  | no CR | 558 | 79 | 1.62 (1.09-2.4) | 0.018 |
|  | Untreated | 327 | 31 | 1.07 (0.65-1.73) | 0.8 |
| Age-recipient |  | 1301 | 146 | 1 (0.98-1.01) | 0.7 |
| Source | BM | 205 | 26 |  |  |
|  | PB | 1096 | 120 | 1.32 (0.83-2.08) | 0.2 |
| TBI | no | 1109 | 128 |  |  |
|  | yes | 192 | 18 | 0.73 (0.43-1.25) | 0.3 |
| Sex-match | Female to male | 353 | 50 |  |  |
|  | Other combinations | 948 | 96 | 0.62 (0.44-0.88) | 0.007 |
| Conditioning | Standard | 512 | 61 |  |  |
|  | Reduced | 789 | 85 | 1.04 (0.71-1.51) | 0.8 |

**Supplementary table 1d- Multivariable analysis of causes of death**

TBI- total body irradiation, CR- complete remission, PTLD- post transplant lymphoproliferative disease, GVHD- graft vs host disease, HSCT- Hematopoietic stem cell transplant
