# Supplementary material for: Comparison of outcomes for HLA-matched sibling and haplo-identical donors in Myelodysplastic syndromes: report from the chronic malignancies working party of EBMT
Source: Blood Cancer J. 2022 Sep 28;12(9):140. doi: 10.1038/s41408-022-00729-y (PMC9515068; doi:10.1038/s41408-022-00729-y)
Supplement: Supplementary file 3 — Supplementary table 3- Multivariate analysis for various factors affecting a- OS, b- NRM for patients > or =40 years of age [file 41408_2022_729_MOESM3_ESM.docx]

|  | Group | HR (95% CI) | p |
| --- | --- | --- | --- |
| Donor (HD vs MSD) | <6m | 1.69 (1.03-2.76) | 0.038 |
|  | >6m | 0.87 (0.5-1.51) | 0.6 |
| Donor age (dec) | <6m | 0.94 (0.75-1.18) | 0.6 |
|  | >6m | 1.15 (0.96-1.4) | 0.14 |
| Donor x Donor age | <6m | 1.03 (0.77-1.37) | 0.9 |
|  | >6m | 0.84 (0.64-1.11) | 0.23 |
| Patient age |  | 1.1 (0.94-1.28) | 0.24 |
| WHO | RA/RARS/del5q/RCMD-(RS) |  |  |
|  | RAEB-1/2 | 0.96 (0.74-1.25) | 0.8 |
|  | Transformed to AML | 1.26 (0.92-1.73) | 0.14 |
|  | Missing | 0.85 (0.58-1.24) | 0.4 |
| Disease status at transplant | CR |  |  |
|  | no CR | 1.28 (1.04-1.57) | 0.022 |
|  | Untreated | 0.92 (0.69-1.22) | 0.6 |
| Source | BM |  |  |
|  | PB | 1.13 (0.86-1.47) | 0.4 |
| CMV serostatus patient | Negative |  |  |
|  | Positive | 1.19 (0.97-1.46) | 0.09 |
| TBI | no |  |  |
|  | yes | 0.99 (0.76-1.29) | 0.94 |
| Sex match | Female to male |  |  |
|  | Other combinations | 0.85 (0.7-1.04) | 0.11 |
| Conditioning intensity | Standard |  |  |
|  | Reduced | 1.01 (0.82-1.23) | 0.96 |

**Overall survival**

|  | Group | HR (95% CI) | p |
| --- | --- | --- | --- |
| Donor (Haplo vs Sib) | <6m | 2.62 (1.46-4.71) | 0.001 |
|  | >6m | 1.12 (0.5-2.54) | 0.8 |
| Donor age (dec) | <6m | 0.87 (0.62-1.2) | 0.4 |
|  | >6m | 0.84 (0.54-1.28) | 0.4 |
| Donor x Donor age | <6m | 1.03 (0.71-1.49) | 0.9 |
|  | >6m | 0.9 (0.58-1.4) | 0.7 |
| Patient age |  | 1.15 (0.93-1.43) | 0.2 |
| WHO | RA/RARS/del5q/RCMD-(RS) |  |  |
|  | RAEB-1/2 | 0.98 (0.68-1.4) | 0.9 |
|  | transformed to AML | 1.11 (0.71-1.75) | 0.6 |
|  | missing | 0.64 (0.36-1.16) | 0.15 |
| Disease status at transplant | CR |  |  |
|  | no CR | 1.34 (0.97-1.84) | 0.078 |
|  | Untreated | 1.5 (1.01-2.22) | 0.043 |
| Source | BM |  |  |
|  | PB | 1.2 (0.82-1.76) | 0.4 |
| CMV serostatus patient | Negative |  |  |
|  | Positive | 1.32 (0.98-1.79) | 0.07 |
| TBI | no |  |  |
|  | yes | 0.76 (0.5-1.15) | 0.2 |
| Sex match | Female to male |  |  |
|  | Other combinations | 0.69 (0.53-0.91) | 0.009 |
| Conditioning intensity | standard |  |  |
|  | reduced | 0.94 (0.7-1.26) | 0.7 |

**NRM- non-relapse mortality**

Supplementary table 2- Multivariate analysis for various factors affecting a- OS, b- NRM for patients > or = 40 years of age
