# Supplementary figures and images for: Comparison of outcomes for HLA-matched sibling and haplo-identical donors in Myelodysplastic syndromes: report from the chronic malignancies working party of EBMT
Source: Blood Cancer J. 2022 Sep 28;12(9):140. doi: 10.1038/s41408-022-00729-y (PMC9515068; doi:10.1038/s41408-022-00729-y)

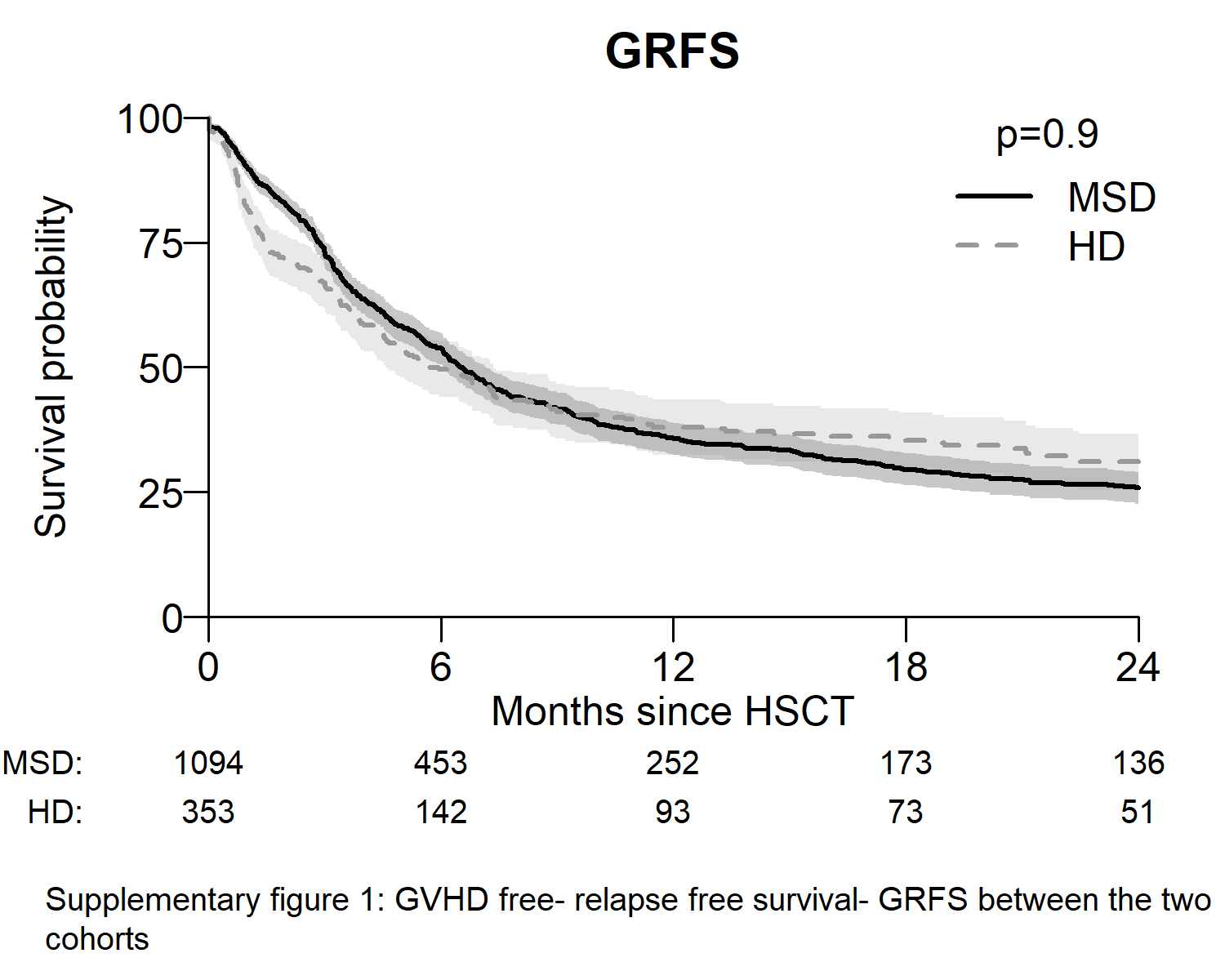

Supplement: Supplementary file 4 — Supplementary figure 1 [file 41408_2022_729_MOESM4_ESM.tif]

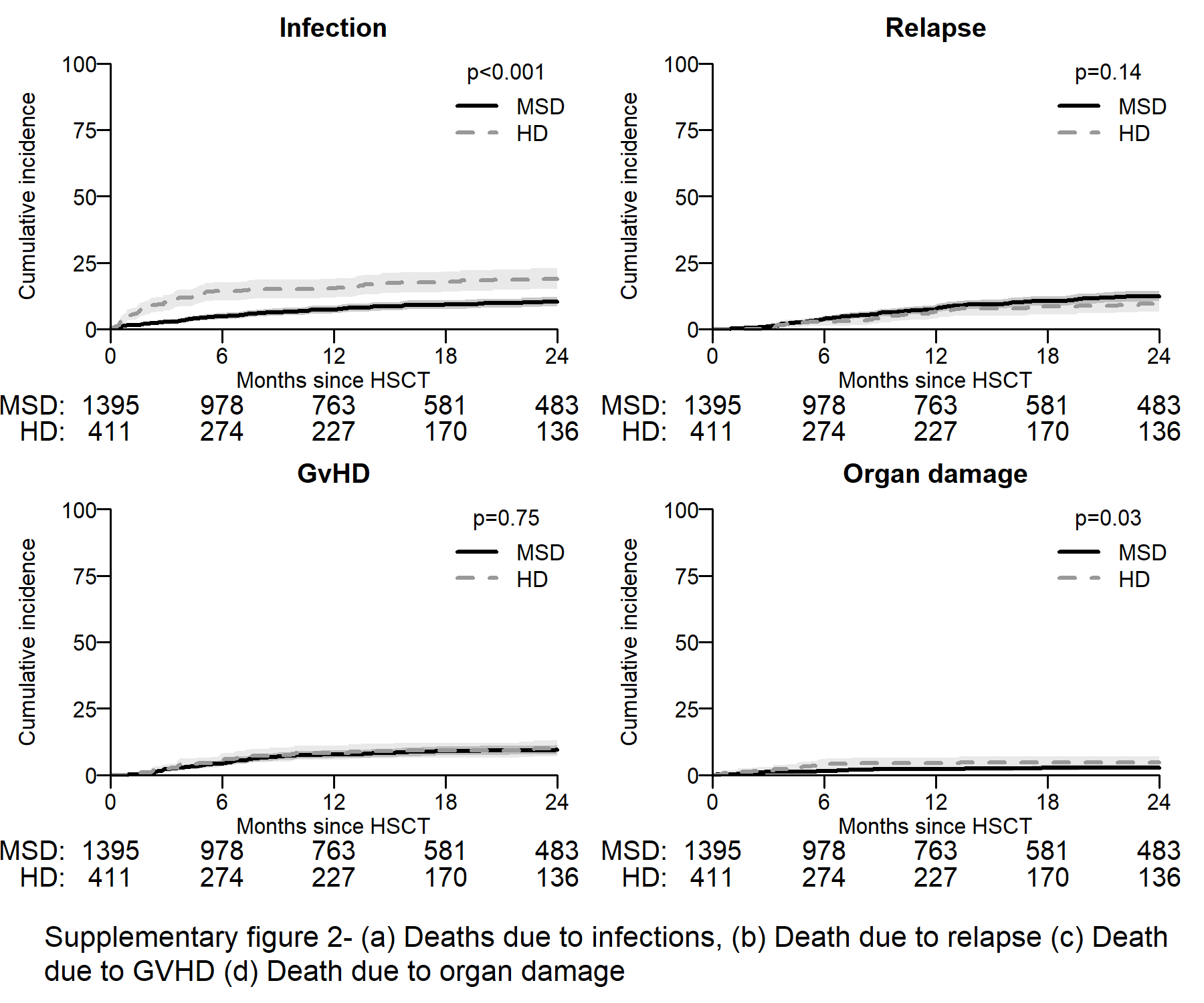

Supplement: Supplementary file 5 — Supplementary figure 2 [file 41408_2022_729_MOESM5_ESM.tif]
